# Peritoneal Administration of a Subunit Vaccine Encapsulated in a Nanodelivery System Not Only Augments Systemic Responses against SARS-CoV-2 but Also Stimulates Responses in the Respiratory Tract

**DOI:** 10.3390/v13112202

**Published:** 2021-11-02

**Authors:** Tuksin Jearanaiwitayakul, Suttikarn Apichirapokey, Runglawan Chawengkirttikul, Jitra Limthongkul, Mathurin Seesen, Phissinee Jakaew, Sakalin Trisiriwanich, Sompong Sapsutthipas, Panya Sunintaboon, Sukathida Ubol

**Affiliations:** 1 Department of Microbiology, Faculty of Science, Mahidol University, Bangkok 10400, Thailand; tuksin.jear@gmail.com (T.J.); suttikarn.api@gmail.com (S.A.); runglawan.cha@mahidol.ac.th (R.C.); jitra.kas@mahidol.ac.th (J.L.); mathurin.jj@gmail.com (M.S.); phissinee.eff@gmail.com (P.J.); 2 Institute of Biological Products, Department of Medical Sciences, Ministry of Public Health, Nonthaburi 11000, Thailand; sakalin.t@dmsc.mail.go.th (S.T.); sompong.s@dmsc.mail.go.th (S.S.); 3 Department of Chemistry, Faculty of Science, Mahidol University, Salaya, Nakornpatom 73170, Thailand; panya.sun@mahidol.ac.th

**Keywords:** SARS-CoV-2 spike glycoprotein, COVID-19 vaccine, adjuvant delivery particles, peritoneal immunization, mucosal immunity

## Abstract

The COVID-19 pandemic has currently created an unprecedented threat to human society and global health. A rapid mass vaccination to create herd immunity against SARS-CoV-2 is a crucial measure to ease the spread of this disease. Here, we investigated the immunogenicity of a SARS-CoV-2 subunit vaccine candidate, a SARS-CoV-2 spike glycoprotein encapsulated in *N*,*N*,*N*-trimethyl chitosan particles or S-TMC NPs. Upon intraperitoneal immunization, S-TMC NP-immunized mice elicited a stronger systemic antibody response, with neutralizing capacity against SARS-CoV-2, than mice receiving the soluble form of S-glycoprotein. S-TMC NPs were able to stimulate the circulating IgG and IgA as found in SARS-CoV-2-infected patients. In addition, spike-specific T cell responses were drastically activated in S-TMC NP-immunized mice. Surprisingly, administration of S-TMC NPs via the intraperitoneal route also stimulated SARS-CoV-2-specific immune responses in the respiratory tract, which were demonstrated by the presence of high levels of SARS-CoV-2-specific IgG and IgA in the lung homogenates and bronchoalveolar lavages of the immunized mice. We found that peritoneal immunization with spike nanospheres stimulates both systemic and respiratory mucosal immunity.

## 1. Introduction

The newly emerging virus named severe acute respiratory syndrome coronavirus 2 (SARS-CoV-2), is an enveloped virus and a member of the Betacoronavirus genus. SARS-CoV-2 contains a positive single-stranded RNA with ~30 kb in length, in which two-thirds of the genome encodes a viral replicase complex, implicated in viral genome synthesis and host immune evasion [1,2,3]. SARS-CoV-2 has spike glycoprotein as a major component of its envelope. The spike, thus, serves as one of the major targets of host immune system [4,5,6]. SARS-CoV-2 is a causative agent of the current global severe respiratory distress syndrome known as coronavirus disease 2019 (COVID-19). Since its emergence in 2019, SARS-CoV-2 introduces at least four waves of the epidemic in various parts of the world [7]. Its epidemics have caused drastically changes to global health infrastructures and severe damages to world economy. Thus, a vital aspect to terminate SARS-CoV-2 epidemic should be implemented. One of these strategies is rapid massive vaccination [8,9].

Different strategies of vaccine development have been applied. The current vaccines available for emergency human use include mRNA, viral vectors and inactivated viruses (https://www.who.int/covid-19/vaccines) (accessed on 18 July 2021), while the promising new subunit vaccine known as Novavax has recently completed a clinical trial Phase III program [10]. Among the vaccine candidates, subunit-based vaccines hold great advantages because of their safety profiles across a broad range of populations. Moreover, the management of this vaccine is easier compared with the more advanced mRNA vaccines in terms of storage and the cold supply chain [11]. Despite its promise, the fact is that subunit vaccines have inherently low immunogenicity. An appropriate adjuvant is an indispensable component of any subunit vaccine to enhance vaccine effectiveness.

The nanodelivery system can serve as an alternative vaccine adjuvant due to its ability to protect, deliver and control the release of immunogens to the right target cells. These activities, in turn, increase the immunogenicity of vaccine candidates. Delivery systems that possess non-toxic, biocompatible and biodegradable properties are significant for medical applications. One example is chitosan nanodelivery particles [12]. This biodegradable compound has a cationic charge which facilitates an electrostatic interaction with the anionic charged molecules on the cellular membrane [13], resulting in enhanced delivery of antigenic materials into the target cells. The application of chitosan or its derivatives has been proven as a delivery system for enhancing the vaccine efficacy of a wide range of antigens. For example, encapsidation of C-terminal truncated dengue virus-2 nonstructural protein 1 (DENV-2 NS1_1-279_) into *N*,*N*,*N*-trimethyl chitosan nanoparticles (NS1_1-279_-TMC NPs) efficiently promoted the uptake of NS1 immunogens by dendritic cells (DCs), leading to activation of these immune cells and potently stimulates functional antibodies and CD8^+^ cells in murine model [14]. Moreover, the encapsidated form of protective antigens (PA) of *Bacillus anthracis* [15,16], inactivated influenza virus [17,18] or recombinant influenza A virus M2e protein genetically fused to the C-terminus of the *Mycobacterium tuberculosis* HSP70 (r4M2e.HSP70c) [19] stimulated robust sterilizing immunity that fully protected animals from mortality and/or reduced the disease burden caused by infection with respective pathogens. The antigenic enhancement properties of TMC delivery systems favor the application of this system in the development of vaccines against infectious diseases.

In the present work, we developed a SARS-CoV-2 vaccine candidate comprising a full-length spike glycoprotein encapsulated within *N*,*N*,*N*-trimethyl chitosan particles (S-TMC NPs). This spike is essential for virus-host recognition and entry [20] and, therefore, serves as a target for anti-SARS-CoV-2 vaccine development. The recombinant spike glycoprotein/S-glycoprotein was derived from *P. pastoris*, purified, and fabricated within TMC NPs. Because we want to investigate the potential of this vaccine candidate to simultaneously stimulate both mucosal and systemic immune responses, thus, vaccine candidate was peritoneally administered to the BALB/c mice. We found that administration via this route activated not only the systemic immune response but also the viral-specific response in the respiratory tract.

## 2. Materials and Methods

### 2.1. Viruses

The wild-type (WT) SARS-CoV-2 (hCoV-19/Thailand/74/2020) and the Delta variant lineage B.1.617.2 (CV-2071) were isolated from the nasopharyngeal swab of confirmed COVID-19 patients at the National Institute of Health, Department of Medical Sciences, Thailand. The genome is completely analyzed by whole genome sequencing to confirm that it is Delta variant or Wuhan virus. Viruses were propagated in Vero cells. The inactivated viruses were prepared via exposure to Ultraviolet-A (UV-A) irradiation for 30 min and were used in the virion-binding ELISA.

### 2.2. Animals

Female BALB/c mice aged 6–8 weeks old were purchased from Nomura Siam International (Nomura Siam International Co., Ltd., Bangkok, Thailand). Animals were housed in an animal facility for a week prior to being subjected to experimentation. All procedures for the animal experiment were performed in accordance with ethical principles and guidelines of the Faculty of Science, Mahidol University–Institutional Animal Care and Use Committee (MUSC–IACUC, protocol number: MUSC63-012-520).

### 2.3. Production of Recombinant SARS-CoV-2 Spike Protein

The SARS-CoV-2 spike protein (S-protein) was produced in *P. pastoris* as previously described [21]. In brief, the S gene (GenBank accession number: NC_045512.2) flanked by the *XbaI* and *KpI* restriction sites encoding the full-length 1273 amino acids of spike protein was codon-optimized and cloned into the pPICαβ expression vector (Invitrogen, Carlsbad, CA, USA). The recombinant DNA was introduced into *P. pastoris* by electroporation. The expressed protein was subsequently purified using affinity chromatography on Ni-NTA agarose (Invitrogen, Carlsbad, CA, USA). The purified protein was evaluated by immunoblotting analysis using mouse anti-His (C-term) antibodies (Invitrogen, Carlsbad, CA, USA) and rabbit anti-SARS-CoV-2 receptor-binding domain (RBD) polyclonal antibodies (Sino Biological, Beijing, China). The SARS-CoV-2 RBD protein used throughout the study was prepared in-house as previously described [22].

### 2.4. Formulation and Characterization of Spike Glycoprotein-Loaded-N,N,N-Trimethyl Chitosan Nanoparticles (S-TMC NPs)

S-TMC NPs were prepared by the ionotropic gelation method as previously described [14]. Briefly, a sodium tripolyphosphate (TPP, 0.167 mg/mL) solution containing S-protein (0.3 mg/mL) and a TMC solution (1 mg/mL) in a HEPES buffer (pH 7.4) with 1% (*v*/*v*) Tween 80 was mixed under continuous stirring for 1 h at room temperature. The S-TMC NPs were separated from unbound S-glycoproteins by centrifugation at 10,000× *g* for 10 min. The pelleted NPs were resuspended in 1× Phosphate-buffered saline (PBS) pH 7.4 (Gibco, Amarillo, TX, USA) for further analysis. The supernatant was collected and used for estimating loading efficiency (LE) by a protein quantitation assay with a Micro BCA protein assay kit (Thermo Fisher Scientific, Rockford, IL, USA). The percentage of LE was determined as previously described [14]. The physical properties of the NPs, including the mean particle size, the polydispersity index (PDI) and their zeta potential were determined by a zetasizer (Malvern Instruments Ltd., Malvern, UK).

### 2.5. Uptake of S-TMC NPs by Phagocytic Cells

Cultures of THP-1 cells, a human leukemic monocytic cell line, were incubated with soluble S-protein (20 μg/mL) or S-TMC NPs containing 20 μg/mL of encapsidated antigen at 4 °C or 37 °C for 4 h. After treatment, cells were harvested, fixed and permeabilized simultaneously with the Cytofix/Cytoperm solution kit (BD Biosciences, San Diego, CA, USA). The permeabilized cells were then incubated with rabbit anti-SARS-CoV-2 RBD polyclonal antibody (1:1000, Sino Biological, Beijing, China), followed by staining with Alexa Fluor 488-conjugated goat anti-rabbit antibody (1:1000, Invitrogen, Carlsbad, CA, USA). The mean fluorescence intensity (MFI) and percentage of Alexa Fluor 488-positive cells were determined by flow cytometry.

### 2.6. Animal Immunization and Specimen Collection

Mice were administered soluble S-protein (S) or S-TMC NPs at 10 or 20 μg S-protein/dose. Mice receiving 1× PBS or empty TMC NPs represented a negative control. The volume of each vaccine dose was 0.1 mL. All animal immunizations were performed via the intraperitoneal route on Days 0, 15 and 30. Blood samples were harvested on Days 14 and 29. All mice were terminated 2 weeks after the booster vaccination (Day 30) and specimens from immunized mice (blood, bronchoalveolar lavage (BAL), lung and spleen) were collected for further analysis.

### 2.7. Antigen-Specific Antibodies Detected Using the ELISA Assay

The titers of SARS-CoV-2 spike or RBD-specific IgG, IgG1, IgG2a and IgA isotype antibodies in specimens of immunized mice were measured using indirect ELISAs. Briefly, 96-well microplates were coated with purified spike or RBD antigens at a concentration of 1 μg/well at 4 °C overnight. The wells were washed with a washing buffer (0.05% Tween-20 in PBS, PBST) and blocked with PBST containing 1% (*w*/*v*) bovine serum albumin (BSA) for 1 h at room temperature. Two-fold serial dilutions of sera, BALs and lung homogenates diluted in a blocking buffer were added to each well (100 μL/well). The reaction between the antigens and the diluted samples was carried out at room temperature for 2 h. After washing, the wells were incubated with the horseradish peroxidase (HRP)-conjugated goat anti-mouse IgG (1:3000, Invitrogen, Carlsbad, CA, USA), IgG1, IgG2a or IgA antibody (1:4000, Southern Biotech, Birmingham, AL, USA) for 2 h. After incubation, the signals were developed by adding the 3,3′,5,5′-Tetramethylbenzidine (TMB) substrate and the reactions were then terminated using a 1 N HCl solution. The optical density (OD) at 450 nm was read using an ELISA reader. The cut-off endpoint titers (EPT) of antigen-specific antibodies were shown as the reciprocal of the highest dilution of samples with an OD value equal to or more than three times the OD value of blank controls [22].

### 2.8. The Virion-IgG ELISA

Microtiter plates were pre-coated with rabbit SARS-CoV-2 RBD-polyclonal antibody (1:4000, Sino Biological, Beijing, China) overnight at 4 °C. The wells were washed with PBST, followed by incubation with 1% BSA (*w*/*v*) in PBST for 1 h at room temperature. After incubation, UV-inactivated SARS-CoV-2 (10^4^ plaque-forming unit (PFU)/well) was added to each well and incubated for 2 h. Wells were washed and then probed with diluted samples for 2 h. After washing, SARS-CoV-2-specific antibodies were detected by the addition of HRP-conjugated goat anti-mouse IgG antibody (1:3000, Invitrogen, Carlsbad, CA, USA).

### 2.9. Antibody Neutralization Assay

Levels of neutralizing antibodies against SARS-CoV-2 were determined by a plaque reduction neutralization assay (PRNT). In brief, mouse sera were heat-inactivated at 56 °C for 0.5 h. The sera were serially diluted four-fold and incubated with an equal volume of 10^2^ PFU of SARS-CoV-2. The virus–antibody mixture was further incubated at 37 °C for 1 h before being inoculated into a monolayer of Vero cells. The plaque assay was then performed as described previously [22]. The neutralizing titers (PRNT_50_) that conferred plaque reduction at 50% were calculated compared with the virus control (virus alone).

### 2.10. Assessment of Vaccine-Induced Cellular Immune Responses

In total, 10^7^ cells/well of the splenic single-cell suspension was stimulated with SARS-CoV-2 spike protein (10 μg/mL) as a specific antigen or Concanavalin A (Con A, 20 μg/mL) as a positive control. The medium-treated splenocytes were a negative control for antigen stimulation. The treated cells were grown at 37 °C for 72 h, and Brefeldin A (BioLegend, San Diego, CA, USA) was added to the culture in the last 5 h of the stimulation period. After treatment, the stimulated cells were harvested and further treated with TruStain FcX (anti-mouse CD16/32 antibody, BioLegend, San Diego, CA, USA). For the analysis of T cell responses, treated cells were stained with antibodies corresponding to CD3, CD4, CD8 and IFN-γ, as described previously [14].

Simultaneously, the culture supernatant was harvested on consecutive days of incubation. The secreted cytokines, including interferon gamma (IFN-γ), interleukin-2 (IL-2) and IL-4, were monitored using an ELISA kit according to the manufacturer’s protocol (BioLegend, San Diego, CA, USA).

### 2.11. Statistical Analyses

Results were presented as means ± standard deviations (SD). Statistical significance between two groups of comparison was analyzed using Student’s *t*-test, where *p* < 0.05 was considered as a significant difference.

## 3. Results

### 3.1. Formulation and In Vitro Characterization of S-TMC NPs

**Figure 1:**
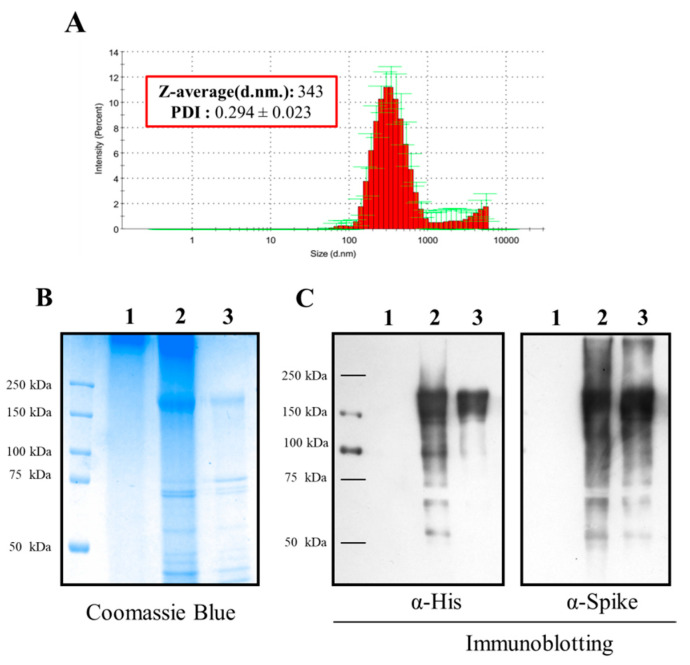
The physical characterization of S-TMC NPs. The ionotropic gelation method was used to encapsulate SARS-CoV-2 spike proteins into TMC NPs. After NP preparation, the mean particle size and polydispersity index (PDI) of the S-TMC NPs were evaluated by a zetasizer (**A**). The entrapment of S-protein in TMC NPs was determined by Coomassie blue staining (**B**) and immunoblotting using the anti-His tag and anti-SARS-CoV-2 RBD antibodies (**C**). 1: Empty TMC NPs; 2: S-TMC NPs; 3: soluble S-antigen.

In the present study, the spike glycoprotein of SARS-CoV-2 was incorporated into TMC NPs by the ionotropic gelation method. After fabrication of the NPs, the S-TMC NPs displayed a colloidal appearance, yielding an average particle size of 343.3 ± 3.4 nm (Figure 1A and Figure S1 and Table S1). The generated NPs were homogenous in size, as indicated by their narrow size distribution (PDI: 0.294 ± 0.023) (Figure S1 and Table S1). The net surface charge of NPs was +14.9 ± 0.451 mV (Table S1), indicating that the surface of S-TMC NPs mainly comprised cationic moieties. The antigen loading efficiency was evaluated and revealed that as much as 95% of the S-protein was loaded into the TMC NPs (Table S1). Moreover, the S-proteins entrapped in TMC NPs could be detected by Coomassie blue staining (Figure 1B). To ensure that the antigenicity of S-proteins remained intact after encapsidation, immunoblotting was performed. As shown in Figure 1C, the encapsidated S-proteins reacted strongly with anti-SARS-CoV-2-specific antibodies. Taken together, these results demonstrated that ionotropic gelation is suitable for generating SARS-CoV-2 spike nanospheres.

### 3.2. Uptake of S-TMC NPs by THP-1 Cells

**Figure 2:**
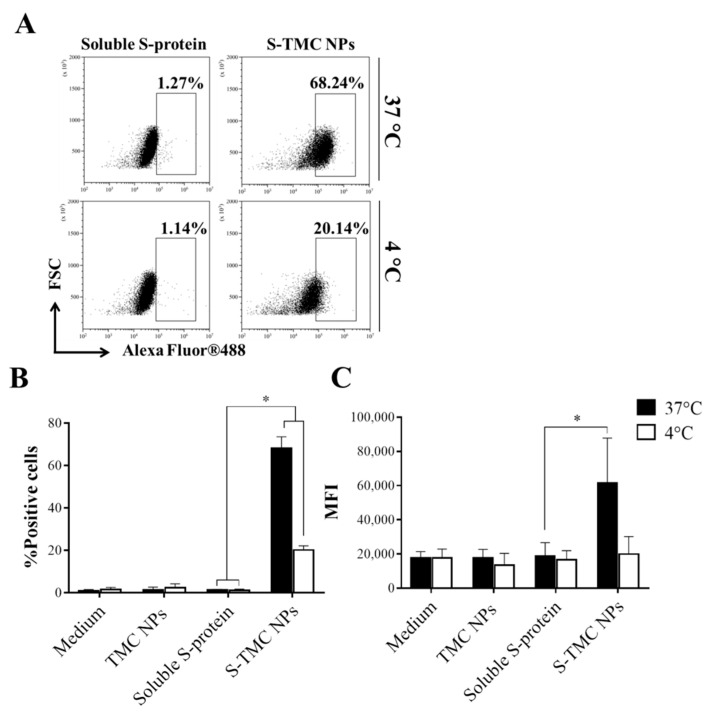
Cellular uptake of S-TMC NPs by phagocytic cells. Cultures of THP-1 cells were incubated with S-TMC NPs (20 μg/mL) or soluble S-protein (20 μg/mL) at 4 °C or 37 °C for 4 h. Intracellular S-antigens were detected by antibody staining with rabbit anti-SARS-CoV-2 antibody and analyzed by flow cytometry; (**A**) dot plot analysis, (**B**) the percentage of S-protein positive cells and (**C**) mean fluorescent intensity (MFI). The results are shown as means ± SD *(n* = 5); * indicates a significant difference between the soluble S-protein and the S-TMC NP treatment (*p* < 0.05).

The particle characteristics, such as size and surface charge, strongly influence the recognition and uptake of immunogens by the antigen-presenting cells (APCs) [23,24]. We evaluated the antigen uptake efficiency of S-TMC NPs by THP-1 cells, a human monocyte cell line. Intracellular staining with SARS-CoV-2-specific antibodies was performed in soluble S-protein- or S-TMC NP-treated cells, followed by analysis using flow cytometry. As shown in Figure 2A,B, 68.24 ± 5.34% of the cells incubated with S-TMC NPs at 37 °C internalized the S-proteins, while only 1.27 ± 0.37% of the soluble S-protein-treated cells engulfed S-antigens. After incubation at 4 °C, 20.14 ± 2% and 1.14 ± 0.65% of the S-protein positive cells were detected in THP-1 cell cultures treated with S-TMC NPs or soluble S-protein, respectively. Significantly, treatment with S-TMC NPs at 37 °C delivered S-proteins more efficiently than treatment at 4 °C (Figure 2C). The results indicated that SARS-CoV-2 S-nanospheres were efficiently internalized into APCs.

### 3.3. S-TMC NPs Strongly Induced the Circulating SARS-CoV-2 Antibody Response

**Figure 3:**
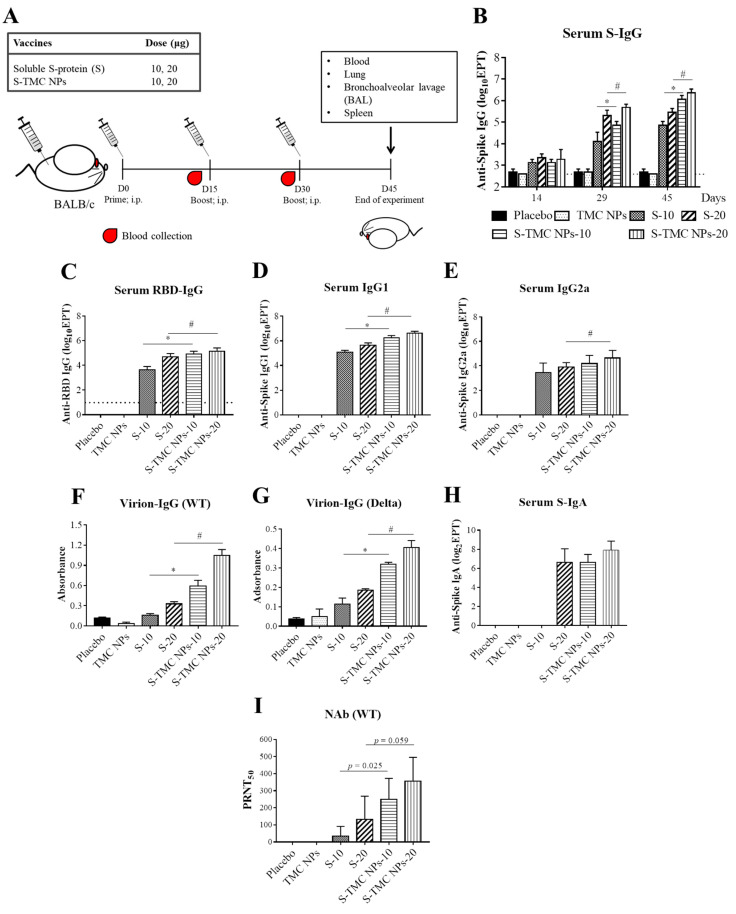
Systemic humoral responses to soluble S-protein or S-TMC NPs. Mice were immunized intraperitoneally with three doses (2 weeks apart) of soluble S-protein (S) or S-TMC NPs (10 or 20 μg/dose). By Day 45 after immunization, samples from immunized mice, including blood, lungs, BALs and spleens, were harvested (**A**). Sera of immunized mice on Days 14, 29 and 45 were subjected to measurement of S-specific IgG by indirect ELISA (**B**). Sera on Day 45 were analyzed for RBD-specific IgG (**C**), S-specific IgG1 (**D**) and IgG2a (**E**) antibody ELISA assays. The levels of SARS-CoV-2-binding antibodies (Virion-IgG) in sera at a dilution of 1:100 were determined by capture ELISA against wild-type (WT) (**F**) and Delta variant viruses (**G**). IgA titers specific to purified S-protein were determined by indirect ELISA (**H**). The titers of serum neutralizing antibodies against SARS-CoV-2 were quantified by PRNT (**I**). Data are presented as means ± SD (*n* = 4); * and # indicate a significant difference between soluble S-protein and S-TMC NPs at 10 and 20 μg/dose, respectively (*p* < 0.05). Dotted line indicates the limit of detection (LOD) of the assay.

We investigated the effect of a S-nanoparticle vaccine candidate on antibody production. Mice were primed with soluble S-protein or S-TMC NPs via intraperitoneal injection, followed by two booster doses on Days 15 and 30 (Figure 3A). The sera of immunized mice were harvested and subjected to IgG titration using indirect ELISA. As depicted in Figure 3B, mice receiving the first dose of soluble S-protein or S-TMC NPs significantly increased production of anti-S IgG antibodies compared to control groups, placebo and TMC NPs, respectively. By Day 29 or two doses of the vaccine, S-TMC NPs induced a greater level of serum anti-S IgG than soluble S-protein. A dose-dependent increase in S-specific IgG titers was observed. The peak in S-specific IgG was sustained until Day 45, the end of study (Figure 3B).

Because RBD is immunodominant and is the target of the neutralizing antibodies in the serum of convalescent COVID-19 patients [5,6], it is of interest to investigate the ability of S-TMC NPs to trigger the production of RBD-specific antibodies. Thus, the IgG response against purified RBD on Day 45 was evaluated by ELISA. A similar pattern of results was found for the RBD-specific IgG antibody response, in which S-TMC NPs stimulated a higher level of anti-RBD IgG than soluble S-protein (5000 ± 3536 vs. 90,000 ± 50,332 and 55,000 ± 30,000 vs. 160,000 ± 113,137 at 10 and 20 µg of soluble and encapsidated S-protein, respectively) (Figure 3C).

To further explore serum IgG responses to S-protein nanosphere immunization, we profiled IgG subtypes in sera obtained on Day 45. The level of IgG2a and IgG1 is widely accepted as an indicative marker of Th-1- and Th-2-mediated responses, respectively. Figure 3D,E revealed that both soluble S-protein- and S-TMC NP-immunized mice robustly elicited anti-S IgG1 and IgG2a production than that of control mice suggesting the induction of both the Th-1 and Th-2 immune responses.

We next determined the ability of serum IgG to recognize native epitopes of SARS-CoV-2 virions using the virion-capture ELISA. As shown in Figure 3F,G, sera from mice immunized with soluble S-antigen or S-TMC NPs reacted with UV-inactivated SARS-CoV-2 particles from both the wild-type and variant strains stronger than did sera from control mice. Interestingly, S-TMC NPs immunized sera contained a higher level of SARS-CoV-2 binding IgG than did sera from soluble S-protein-immunized mice. As expected, the antibodies induced by our vaccine candidate bound to Wuhan strain stronger than to the Delta variant (Figure 3F,G). The results suggest that the S-protein-specific antibodies conferred by S-TMC NP immunization may have a broad neutralizing capacity due to their capability to interact with both the Wuhan isolate of SARS-CoV-2 (hCoV-19/Thailand/74/2020) and the Delta variant of the virus.

IgA is the antibody class produced in the largest quantities by the body. The predominant form is the dimeric IgA found in mucosal secretions, while the less abundant monomeric form is primarily found in serum [25]. Serum IgA has been detected in COVID-19 patients and appears to be detectable earlier than IgM or IgG antibodies [26,27]. Therefore, we determined the levels of the specific serum IgA response to S-protein and RBD antigens in the sera of immunized mice on Day 45. Figure 3H showed that anti-S IgA antibodies were strongly induced in all mice immunized with 20 μg of soluble S-protein or 10 or 20 μg of S-TMC NPs. In contrast, only one out of four mice that received a low dose (10 μg) of soluble S-protein developed serum anti-S IgA at 1:25 dilution while the other three mice showed undetectable level of anti-S IgA (data not shown in Figure 3H). In addition, 20 μg S-TMC NPs activated anti-S IgA production stronger than that soluble S-protein did at the same concentration (131 ± 85 vs. 275 ± 150 at 20 µg of soluble and encapsidated S-protein, respectively).

For RBD-specific IgA production, 2/4 of mice immunized with 10 μg/dose of S-TMC NPs elicited an RBD-specific IgA response at 1:25 dilution, while only one out of four mice received 20 μg/dose of S-TMC NPs developed a detectable level of RBD-specific IgA at 1:25. None of mice that received soluble S-proteins produced a detectable level of RBD-IgA (data not shown). We also determined whether serum IgA could interact with native epitopes of SARS-CoV-2 virions. The sera were subjected to the virion-capture ELISA. Unfortunately, we were unable to detect SARS-CoV-2-specific IgA antibodies in all groups of immunized mice (data not shown). This is contrary to the anti-SARS-CoV-2 IgG antibody stimulation shown above. This may be due to much lower amounts of IgA being found on Day 45 compared with IgG antibodies (2,457,600 ± 945,931 vs. 275 ± 150 for anti-S IgG and IgA antibodies at 20 µg of the encapsidated immunogen, respectively).

To investigate whether the generated S-protein-specific antibodies could neutralize the viral infection, the levels of neutralizing antibody (NAb) in immunized sera obtained on Day 45 were determined by an in vitro neutralization assay (PRNT). Mice receiving either soluble S-protein or the particulate form of S-protein generated neutralizing antibodies against wild-type SARS-CoV-2 (Figure 3I). Among the groups of immunized mice, the S-TMC NP group had higher titers of SARS-CoV-2 neutralizing antibodies than did those receiving the soluble form of S-protein at the same concentration. The NAb titers were 250 ± 122 and 357 ± 140 for 10 and 20 μg/dose of encapsidated S-protein, respectively (Figure 3I). Collectively, our results suggest that S-TMC NPs activate potent systemic production of antibodies with antiviral neutralizing activity against SARS-CoV-2.

Our investigation of serum IgG and IgA production indicated that both soluble S-protein and S-TMC NPs were effective in inducing systemic humoral immunity. Notably, encapsidated S-protein was more immunogenic than the soluble form of S-protein, which was supported by its ability to activate robust systemic antibody production. Moreover, the serum immunoglobulins (IgG and IgA) showed a dose-dependent anti-SARS-CoV-2 neutralizing activity profile. This confirmed the advantage of the nanodelivery system for enhancing the antigenicity of the SARS-CoV-2 subunit vaccine.

### 3.4. Immunization with S-Protein Nanoparticles Induced Systemic Cell-Mediated Immunity

**Figure 4:**
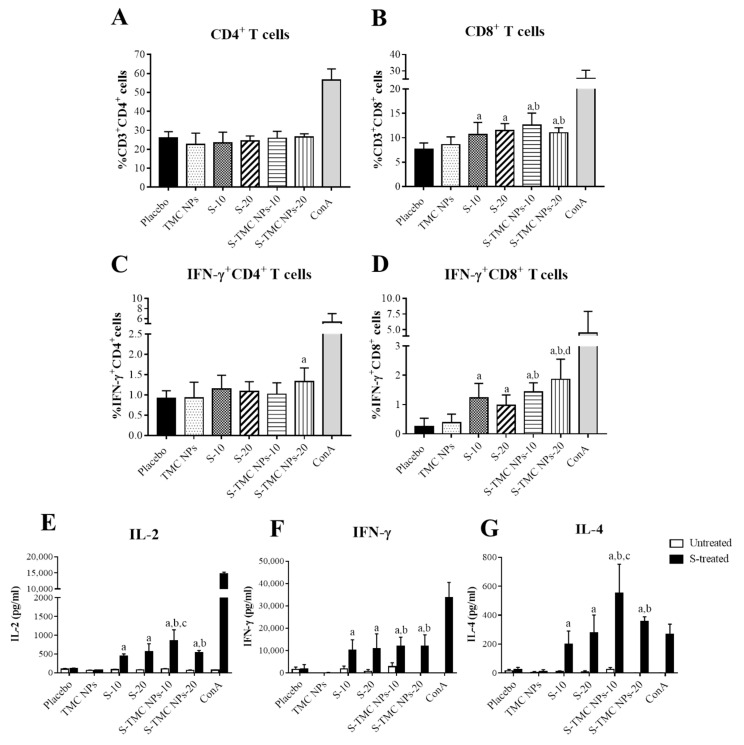
Cellular immune responses to soluble S-protein or S-TMC NPs. Splenocytes of immunized mice that received three doses of soluble S-protein or S-TMC NPs were isolated and stimulated ex vivo with 10 μg/mL of S-antigens for 72 h. Frequencies of S-specific CD4^+^ (**A**), CD8^+^ (**B**), IFN-γ^+^CD4^+^ (**C**) and IFN-γ^+^CD8^+^ (**D**) T cells in cultures of stimulated splenocytes were monitored by antibody staining, followed by analysis using flow cytometry. The levels of splenic cytokines, including IL-2 at 24 h (**E**), IFN-γ at 72 h (**F**) and IL-4 at 72 h (**G**) after antigen stimulation, were quantitated by ELISA. The results are presented as means ± SD (*n* = 4); “a” indicates significant differences between placebo and soluble S-protein or S-TMC NPs. “b” indicates significant differences between TMC NPs and S-TMC NPs. “c and d” indicate significant differences between soluble S-protein and S-TMC NPs at 10 and 20 μg/dose, respectively (*p* < 0.05).

To assess the T cell response activated by our vaccine platform, mice were vaccinated intraperitoneally with either soluble S-protein or S-TMC NPs, as described in the Materials and Methods section. Spleens were harvested at 2 weeks after the last booster shot. Splenocytes were cultured and stimulated with S-proteins. T cell profiles, including the percentages of CD4^+^ or CD8^+^ T cells and activated T cells (the percentages of IFN-γ^+^CD4^+^ and IFN-γ^+^CD8^+^ T cells), were evaluated by flow cytometry. The results showed that immunization with either soluble S-protein or S-TMC NPs increased the frequency of CD8^+^ T cells but not CD4^+^ T cells in the spleens of immunized mice (Figure 4A,B and Figure S2). Despite the frequency of CD4^+^ T cells in the groups of immunized mice being unaffected, S-TMC NPs at a high dose (20 μg) significantly activated IFN-γ^+^CD4^+^ T cell population compared to the placebo group but not other groups (Figure 4C). Furthermore, mice receiving either soluble S-protein or S-TMC NPs elicited strong IFN-γ^+^CD8^+^ T cell response, in which the greatest frequencies of activated CD8^+^ T cells were demonstrated in mice immunized with S-TMC NPs at 20 μg/dose (Figure 4D and Figure S2). These results suggested that the SARS-CoV-2 spikes in nanoparticle forms are highly immunogenic to the IFN-γ^+^CD8^+^ cell response but less efficient in stimulation of IFN-γ^+^CD4^+^ cells.

We further validated the vaccine-induced cellular immune response. The levels of secreted cytokines (IL-2, IFN-γ and IL-4) in a culture supernatant of antigen-treated splenocytes were quantitated by ELISA. As shown in Figure 4E–G, splenocytes isolated from soluble S-protein- or S-TMC NP-immunized mice strongly responded to ex vivo stimulation with SARS-CoV-2 spike antigens by upregulation of IL-2, IFN-γ and IL-4 production. Splenocytes from these groups of immunized mice produced a significantly higher level of IL-2, IFN-γ and IL-4 compared with the diluent control or TMC NP-treated mice. Overall, the upregulation of splenic cytokine production and the activation of effector T cells suggested that soluble S-protein and S-TMC NPs robustly stimulated the cellular immune response.

### 3.5. Intraperitoneal Administration of S-TMC NPs Stimulated Mucosal Immunity in the Respiratory Tract

**Figure 5:**
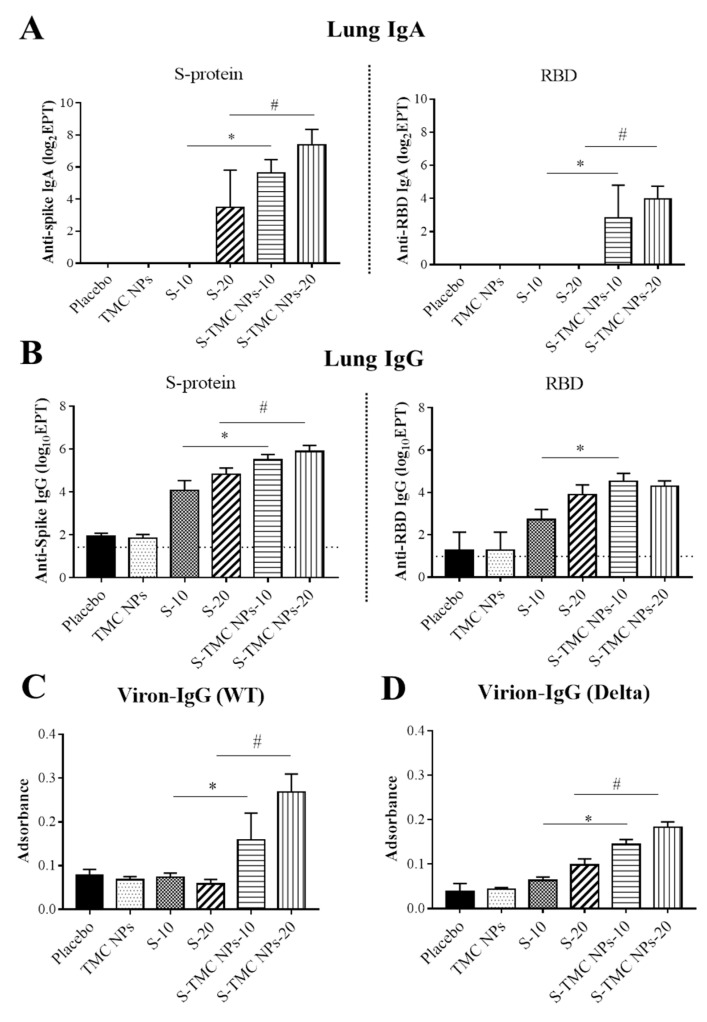
Mucosal responses to soluble S-protein or S-TMC NPs in the lungs of immunized mice. Lungs of immunized mice were collected on Day 45 and processed by homogenization. The levels of S- and RBD-specific IgA (**A**) or IgG (**B**) antibodies in lung homogenates were detected by indirect ELISA. Binding antibodies against SARS-CoV-2 particles (virion–IgG) of WT (**C**) and Delta variant viruses (**D**) present in the lung homogenates diluted at 1:100 was determined by capture ELISA. Data are means ± SD (*n* = 4); * and # indicate a significant difference between soluble S-protein and S-TMC NPs at 10 and 20 μg/dose, respectively (*p* < 0.05). Dotted line indicates the LOD of the assay.

Mucosal immunity serves as a host immune sentinel that protects mucosal tissues from invading pathogens such as SARS-CoV-2 [28,29]. Because SARS-CoV-2 primarily targets cells of the respiratory tract, as such, it is of great interest to see the potential effects of our vaccine candidate on the activation of respiratory mucosal immunity through the intraperitoneal route of administration. Mice were peritoneally immunized with three doses of the vaccine candidate as described in the Materials and Methods section. On Day 45, the lungs of immunized mice were harvested. To obtain BAL, harvested lungs were flushed with 1 mL of 1× PBS. The harvested fluid, containing antibodies present in the bronchioalveolar cavity, was subjected to antibody quantitation. Apart from antibodies present in airway spaces, the lung tissues (interstitial tissue) also contain a certain amount of antibodies and antibody-secreting cells, especially memory-resident B cells. These immune players can quickly respond to and minimize the effects of respiratory infections [30]. Based on this information, the IgA and IgG antibodies that are present in the lung tissue were investigated. The flushed lungs were homogenized and subjected to IgA, and IgG quantitation using an indirect ELISA. Figure 5A shows that IgA responses against purified S- and RBD antigens were detected in the lungs of mice that were administered soluble S-protein or S-TMC NPs. S-TMC NP-immunized mice developed a robust lung IgA response against S-protein and moderate IgA levels against RBD antigens at a 1:10 dilution of lung homogenates. In contrast, soluble S-protein at 20 µg, but not 10 µg, was able to stimulate moderate levels of anti-S IgA antibodies but could not promote RBD-specific IgA production (Figure 5A). To see whether lung IgA could recognize epitopes on intact SARS-CoV-2 particles, a virion-capture ELISA was performed. Unexpectedly, the lung homogenates of immunized mice contained an undetectable level of anti-SARS-CoV-2 IgA antibodies (data not shown).

The presence of lung IgG was also investigated in our study. Typically, this class of antibody enters respiratory secretions both nonspecifically and specifically. It is possible that high levels of circulating IgG may slowly flow from the high concentration in the blood out into the lower depths of the lungs, where junctions between the cells are leaky [31]. Alternatively, neonatal Fc receptor (FcRn), which is selectively expressed on the respiratory epithelium, transports IgG across the mucosal barriers [32,33]. This may explain how antibodies induced via the intramuscular route of vaccination protect us from severe infections and hospitalization. We therefore determined the level of IgG in lung homogenates using an indirect ELISA against S-protein and RBD. As shown in Figure 5B, administration with soluble S-protein or S-TMC NPs result in a significant increase in lung IgG specific to S-protein and RBD compared to control mice. In particular, mice immunized with S-TMC NPs had higher levels of S- and RBD-specific IgG in their lungs compared to mice that received soluble S-protein. In addition, lung IgG from S-TMC NP-immunized mice bound strongly to structural epitopes on SARS-CoV-2 particles derived from the wild-type strain as well as the Delta variant (Figure 5C,D).

**Figure 6:**
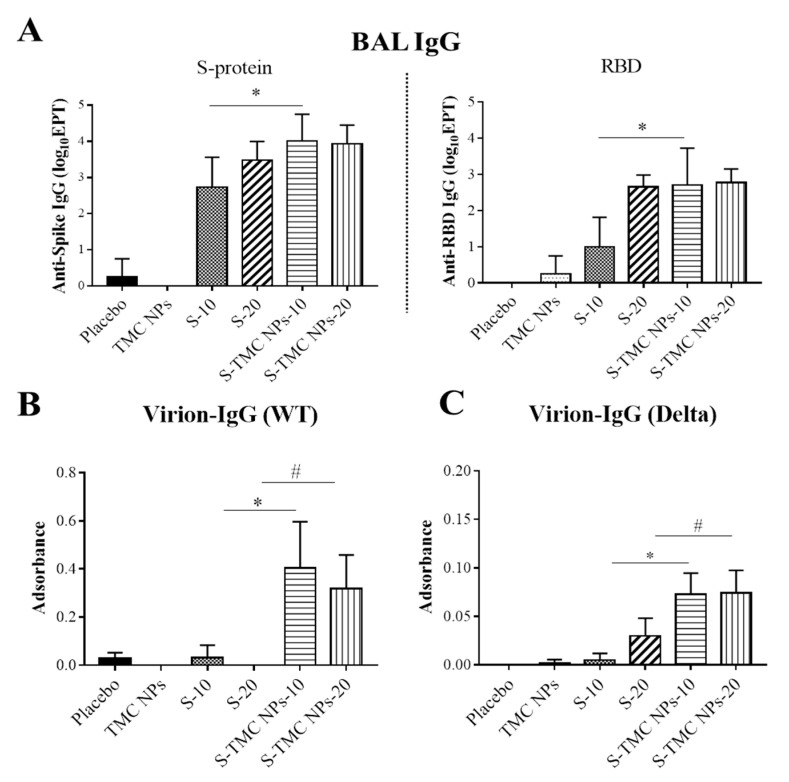
Mucosal responses to soluble S-protein or S-TMC NPs in the BALs of immunized mice. BALs were harvested from immunized mice on Day 45 and subjected to quantitation of anti-S and anti-RBD IgG antibodies using indirect ELISA (**A**). BALs diluted at 1:10 were analyzed for virion–IgG capture ELISA assays against WT SARS-CoV-2 (**B**) and the Delta variant strain (**C**). The results are shown as means ± SD (*n* = 4); * and # indicate a significant difference between the mice immunized with soluble S-protein and S-TMC NPs at 10 and 20 μg/dose, respectively (*p* < 0.05).

We also assessed the presence of IgA and IgG in BALs. The IgA and IgG in BALs of immunized mice on Day 45 were determined by an indirect ELISA. We found that only one out of four mice immunized with 20 μg/dose of soluble S-protein generated a detectable level of IgA specific to S-protein in BALs diluted at 1:8. However, 50% of mice treated with 10 µg or 20 µg S-TMC NPs elicited S-specific IgA response at 1:8 dilution (data not shown). Unexpectedly, none of the peritoneally immunized mice produced a detectable level of RBD-specific IgA in BALs (data not shown). In contrast, increased levels of both S-protein and RBD-specific IgG were found in the BALs of mice upon peritoneal immunization with either soluble S-protein or S-TMC NPs. Mice immunized with 20 µg/dose of either S-protein or S-TMC NPs produced S- and RBD-specific IgG at similar level (Figure 6A). The difference in S- and RBD-specific IgG production was found in mice immunized with a low dose of immunogens in which the S-TMC NPs at 10 µg/dose were more immunogenic than S-protein at the same concentration. Another interesting point was revealed when these antibodies were tested against the native configuration of immunogens through virion capture ELISA. The stronger responses of anti-virion IgG against the wild-type virus and the Delta variant were detected in BALs obtained from S-TMC NP-immunized mice compared to mice received soluble S-protein (Figure 6B,C).

Taken together, an increase in the total antibody responses was found in the lungs of mice peritoneally immunized with either soluble S-protein or S-TMC NPs. In particular, S-TMC NPs that mounted a significant mucosal immune response indicate that, somehow, antigens taken up by the peritoneal mucosal tissues can provoke immune responses in the respiratory tract.

## 4. Discussion

In our present study, a carbohydrate nanodelivery system was used to develop an S-protein nanoparticle vaccine candidate. This used an electrostatic conjugation system to package spike glycoproteins of SARS-CoV-2 into TMC NPs. This encapsidation method is simple whilst alleviating the risk of antigen instability during the preparation process that could easily be scaled up further in response to mass vaccination campaigns. The resulting NPs displayed desirable characteristics for presenting S-protein epitopes and optimizing antigen delivery efficiency. This was shown by not only an increase in the number of S-protein-positive cells but also the amount of S-protein per cell in cultures of S-TMC NP-treated THP-1 cells (Figure 2A–C).

We then investigated the potential of the vaccine candidate to stimulate the immune response using an in vivo model. S-TMC NP-immunized mice elicited stronger systemic antibody responses, including total IgG, IgG1, IgG2a and IgA, against the spike antigens (S-and RBD proteins) of SARS-CoV-2 than did soluble S-protein administration. More interestingly, S-TMC NPs were effective at eliciting antibodies capable of recognizing complex epitopes on virions of the wild-type virus as well as the Delta variant, which is the current strain circulating globally, indicating that this is a good immunogen platform. The utility of TMC nanoparticles as a delivery system has also been strengthened for improving vaccine efficacy against outer membrane protein-1 (Omp-1) [34], pertussis toxoid (PTd) and filamentous hemagglutinin (FHA) proteins (Ptd + FHA) [35], inactivated PR8 influenza virus [36], PA antigen [15,16], DENV-2 NS1_1-279_ [14] and UV-inactivated dengue virus-2 [37]. These observations may be due to the fact that TMC has inherent immunomodulatory effects that enhance antibody responses [38]. Furthermore, we showed here that sera obtained from S-TMC NP-immunized mice have an antiviral activity, which was detected via inhibition of SARS-CoV-2 infection in an in vitro assay. Notably, the level of neutralizing activity correlated positively with the levels of anti-spike protein, anti-RBD and anti-SARS-CoV-2 virion IgG antibodies, which were detected by ELISA. Currently, neutralizing antibody levels are a predictive marker of SARS-CoV-2 immune protection [39,40]. Therefore, the production of potent virus-neutralizing antibodies induced by this vaccine platform may confer protection against SARS-CoV-2 in challenge experiments, which are under investigation.

Besides the antibodies, a cell-mediated immune response is recognized as a powerful weapon of host immunity against viral pathogens. Though the protective roles of T cells in SARS-CoV-2 infection remain elusive, clinical data have revealed that dysregulation of T cell responses has been observed in patients with severe diseases [41]. On the other hand, an increase in the SARS-CoV-2-specific CD8^+^ cell population, which is crucial for the clearance of viral infections, was reported in the peripheral blood mononuclear cells (PBMC) of convalescent patients with mild symptoms [42]. Moreover, SARS-CoV-specific CD8^+^ cells have also been demonstrated to provide protection against lethal challenge with SARS-CoV in mice [43]. Our present findings demonstrated that immunization with soluble S-protein or S-TMC NPs stimulated a decent level of IFN-γ^+^CD8^+^ cell responses but much less for IFN-γ^+^CD4^+^ cell response. Specifically, IFN-γ^+^CD8^+^ cells were greatly generated following S-TMC NP vaccination. As a result, it would be worth conducting further studies to determine whether the spike-specific cytotoxic T lymphocyte (CTL) response induced by our vaccine platform would have an eradicating action against SARS-CoV-2-infected cells. The mechanism by which S-TMC NPs enhance the CTL response may be involved in endosomal escape. This would enable the encapsidated antigens to be further targeted by the major histocompatibility complex class I (MHC-I) molecules [44].

Most of the current SARS-CoV-2 vaccines for emergency use are administered through intramuscular injection [45]. As SARS-CoV-2 is known to be transmitted mainly via respiration, the major target cells of SARS-CoV-2 are cells of the respiratory system. Therefore, it is imperative to understand the most effective means of systemic vaccination for activating local mucosal immunity. Typically, it is widely accepted that systemic immunization has a very low impact on the mucosal immune response. This is supported by previous reports that intramuscular administration with adenovirus-based or alum-adjuvanted inactivated SARS-CoV-2 vaccines was unable to afford sterilized protection against nasal virus replication [29,46]. Nonetheless, some antigens combined with adjuvants have been reported to induce specialized local immunity. For instance, intraperitoneal immunization with poly-(I:C) adjuvanted inactivated SARS-CoV strongly elevated antigen-specific IgA and IgG at multiple sites of the mucosa [47]. More recently, delivery of poly (lactic-co-glycolic acid) nanoparticles (PLGA NPs) containing the recombinant major outer membrane protein (rMOMP) of *C. muridarum* to mice via subcutaneous injection efficiently protected mice from genital bacterial challenge by inducing strong IgG but low IgA responses in the mucosal tissues [48]. In line with these reports, we revealed here that the mucosal antibody responses were markedly augmented in the lungs and BALs of mice peritoneally immunized with S-TMC NPs. Both anti-SARS-CoV-2 IgA and IgG were found in lung homogenates and BALs. This indicated that our vaccine platform, given through the intraperitoneal route, not only stimulated IgA production in the respiratory tract but also translocated IgG from the circulation to the lungs. Additionally, antibodies provided by S-TMC NPs stimulation were more efficient in recognizing structural epitopes on virion surface compared to antibodies produced in response to soluble S-protein stimulation. This evidence suggests us that encapsidation of S-protein into TMC NPs may, somehow, facilitate the S-protein folding into a structure close to the native structure present on SARS-CoV-2 particles.

IgA responses have already been described as playing a role in immune responses to SARS-CoV-2. For example, serum IgA has been detected in COVID-19 patients and appears to be detectable earlier than IgM or IgG antibodies [26,27]. This suggests that IgA may be the first antibody type that encounters the SARS-CoV-2 invader. How IgA participates in protection against SARS-CoV-2 infection is unclear. In general, the secretory IgA (sIgA) has a number of essential functions within mucosal immunity, such as preventing host cell infection via immune exclusion, contributing to viral clearance via the agglutination and shielding of microbial adhesins for later clearance via ciliary activity [49]. In the case of SARS-CoV-2, sIgA antibodies may prevent an interaction between SARS-CoV-2 spike proteins and angiotensin-converting enzyme 2 (ACE2) on epithelial target cells [50,51]. Beyond its role in immune exclusion, sIgA can initiate and regulate the process of myeloid immune responses through the Fc fragment of IgA receptor (FcaR) to the IgA Fc region that is found on multiple immune and epithelial cells, resulting in a broad range of effector functions involving both humoral and cellular responses [49]. Alongside the sIgA response, IgG is a prominent class of antibodies present in the mucosal linings’ surfaces and secretions. IgG cooperates with IgA as part of the local immune defense in airway spaces against microbial pathogens [52]. It has been reported that an increase in mucosal IgG levels is correlated with a lower viral load during respiratory syncytial virus (RSV) infection [53]. IgG acts in a similar manner to IgA by neutralizing the free virus particles or selectively opsonizing virus particles, which are then eliminated by Fc fragment of IgG receptor (FcγR)-bearing cells [54]. Intracellularly, the IgG–virus complex mediates the host defense to infected cells [55,56]. We speculate that the anti-SARS-CoV-2 IgG and IgA revealed in our model may help to reduce the spread of viruses from the nasal tissue to lungs, thus decreasing disease severity.

It is unclear how systemic immunization with S-TMC NPs stimulates mucosal immunity. We hypothesized that TMC may facilitate antigen diffusion from the inoculation site into the regional draining lymph nodes (DNs) lining the peritoneal cavity. At the DNs, the encapsidated antigen will be captured by the local APCs and drive APC maturation. These activated APCs then migrate to the mucosa-associated lymphoid tissues (MALTs) of the respiratory airway, where they present the antigen to naïve T cells, resulting in activation of the mucosal immune response. Alternatively, TMC may enhance uptake by local APCs (resident macrophages, DCs and B cells) at the site of vaccine administration. Finally, these cells migrate to MALTs and present antigens to T cells.

Despite the promising results of this study, there are a few limitations that need to be addressed. These include challenge studies with susceptible host models to confirm the extent of vaccine protection, the neutralizing capacity of antibodies against other SARS-CoV-2 variant strains and the durability of protective immunity. These concerns are worth further investigation.

In summary, we explored the advantage of a biodegradable nanodelivery system, TMC nanodelivery particles, for enhancing the immunogenicity of the spike protein of SARS-CoV-2. The systemic administration of this vaccine platform in BALB/c mice showed its superior induction of the immune response both systemically and in the mucosal areas over the soluble form of the antigen. Furthermore, our vaccine platform is feasible for future adaptability to the global response against the ongoing SARS-CoV-2 pandemic of the variants as well as other newly emerging viruses. Indeed, these findings provide not only a novel platform for SARS-CoV-2 vaccine candidates but also additional evidence that intraperitoneal vaccination confers mucosal immunity at the respiratory tract. This will give the high benefit to the world population if one form of vaccine effectively protects us through various strategies of vaccination.

## Data Availability

The data presented in this study are contained within the article.

## Supplementary Materials

The following are available online at https://www.mdpi.com/article/10.3390/v13112202/s1, Table S1: Physical properties of S-TMC NPs, Figure S1: Size distribution of S-TMC NPs by dynamic light scattering, Figure S2: Gating strategy used for quantitation of splenic T lymphocytes.
