# Supplementary material for: Peritoneal Administration of a Subunit Vaccine Encapsulated in a Nanodelivery System Not Only Augments Systemic Responses against SARS-CoV-2 but Also Stimulates Responses in the Respiratory Tract
Source: Viruses. 2021 Nov 2;13(11):2202. doi: 10.3390/v13112202 (PMC8617950; doi:10.3390/v13112202)
Supplement: Supplementary file 1 [file viruses-13-02202-s001.zip › viruses-1391776-supplementary.pdf]

Supplement data

Table S1 Physical properties of S-TMC NPs

Nanoparticles	Particle size (nm)	Polydispersity index (PDI)	Zeta potential (mV)	% loading efficiency (LE)
TMC NPs	358.1 ± 7.404	0.354 ± 0.023	15.7 ± 0.551	-
S-TMC NPs	343 ± 3.402	0.294 ± 0.023	14.9 ± 0.451	95.08 ± 4.50

Data are shown as mean ± SD of three independent experiments.

Figure S1

			Size (d.nm):	% Intensity:	St Dev (d.n...
Z-Average (d.nm):	341.7	Peak 1:	350.8	92.3	142.1
PdI:	0.279	Peak 2:	4601	7.7	827.9
Intercept:	0.939	Peak 3:	0.000	0.0	0.000
Result quality :	Good				

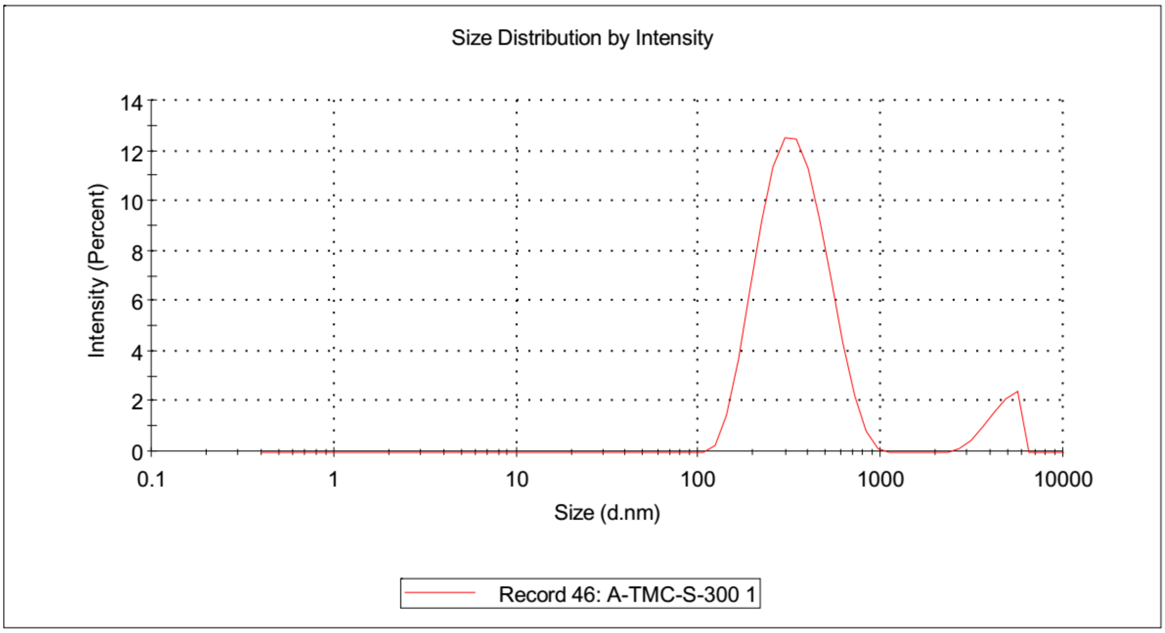

Figure S1 Size distribution of S-TMC NPs by dynamic light scattering. Data presents a representative experiment from three independent sets of data.

Figure S2

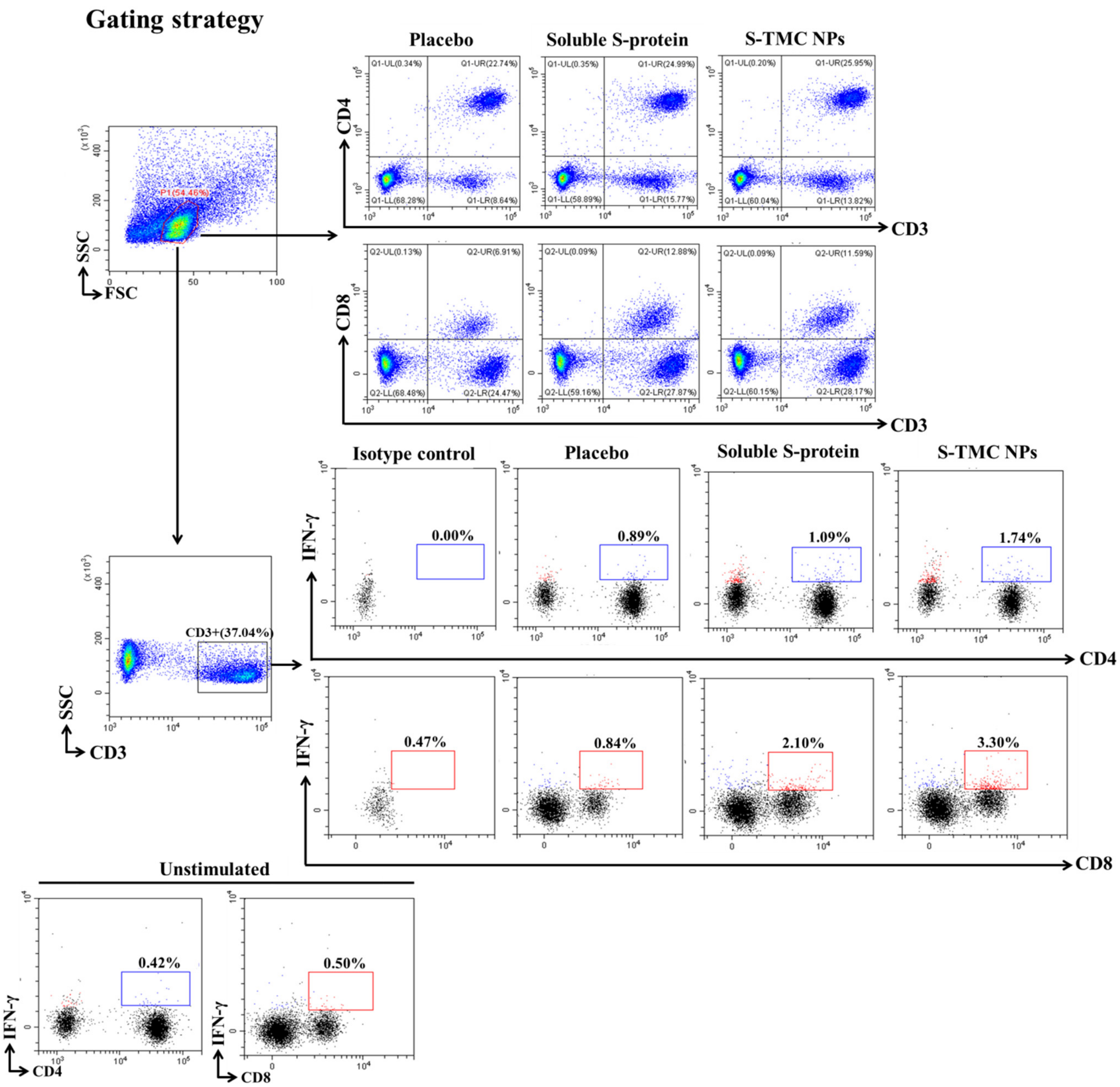

Figure S2 Gating strategy used for quantitation of splenic T lymphocytes. The cultivated splenocytes were *ex vivo* stimulated with 10 µg/mL of recombinant S-protein for 72 h. After treatment, stimulated cells were harvested and stained with antibodies specific to CD3, CD4,

CD8 and IFN-γ. The percentages of $CD3^+CD4^+$, $CD3^+CD8^+$, IFN-γ^+CD4^+ and IFN-γ^+ $CD8^+$ cells in cultivated splenocytes were determined by flow cytometry.
